# Efficient feeder cells preparation system for large-scale preparation and application of induced pluripotent stem cells

**DOI:** 10.1038/s41598-017-10428-5

**Published:** 2017-09-25

**Authors:** Pengdong Li, Shichao Wang, Lixiang Zhan, Xia He, Guangfan Chi, Shuang Lv, Ziran Xu, Yuhan Xia, Shuzhi Teng, Lisha Li, Yulin Li

**Affiliations:** 1 The Key Laboratory of Pathobiology, Ministry of Education, Norman Bethune College of Medicine, Jilin University, Changchun, China; 2 Department of Gynecology, Third Hospital of Jilin University, Changchun, China

## Abstract

Despite recent progress in the preparation of feeder cells for human induced pluripotent stem cells (hiPSCs), there remain issues which limit the acquisition of feeder cells in large scale. Approaches for obtaining feeder cells quickly on a large scale are in immediate need. To reach this goal, we established suspension-adhesion method (SAM) and three-dimensional (3D) suspension method (3DSM). In SAM, mouse embryonic fibroblast (MEF) growth were fully inhibited by 10 μg/ml mitomycin-C (MMC) in 0.5 hours, and the feeder cells generated display higher adherent and recovery rates as well as longer survival time compared to conventional method (CM). 3DSM, an optimized method of SAM in which MEFs were cultured and MMC treated in suspension, was developed to lower the costs and workload using CELLSPIN System. The yield of feeder cells is several times the yield of SAM while the adherent and recovery rates and the capacity of supporting hiPSCs growth were not sacrificed. Hence, 3DSM is an economical and easy way to generate large-scale feeder cells for hiPSCs cultures.

## Introduction

Induced pluripotent stem cells (iPSCs) can be obtained from somatic cells by forced expression of a defined set of reprogramming factors, including either the combinations of Oct4, Klf4, Sox2, and c-Myc, or of Oct4, Sox2, Nanog, and Lin28^1–4^. We previously reported to obtain iPSCs from human hair follicles-mesenchymal stem cells (hHF-MSC-derived iPSCs) using four Yamanaka factors (Oct4, Sox2, c-Myc and Klf4)^5^. These iPSCs are capable of self-renewal and differentiate into various cell types, feeder cells are required to support their growth while maintaining pluripotency.

Feeder cells are known to produce growth factors, adhesion molecules, and extracellular matrix. The most widely used feedder cells include mouse embryonic fibroblasts (MEFs). Recently, a xeno-free cell culture method was established to avoid contamination by pathogens and animal proteins^6,7^. In that system, mouse feeder cells are replaced with human cells such as human fetal and adult fibroblasts^8^, human fetal muscle fibroblasts^9^, foreskin fibroblasts^10^, amniotic mesenchymal cells^11^, adipose-derived mesenchymal stem cells^12^, bone marrow mesenchymal stem cells^13–15^, placenta-derived mesenchymal stem cells^16^, multipotent mesenchymal stem cells of desquamated endometrium^17^, and decidua-derived mesenchymal cells^18^.

In spite of recent progress in hiPSCs culture conditions, large-scale production of hiPSCs by robust and economical methods has been one of the major challenges for the translational realization of hiPSCs technology^19^. To achieve large-scale production of hiPSCs, a large-scale culture system for hiPSCs expansion using the E8 chemically defined and xeno-free medium has recently been developed^20^. However, the efficiency of human feeder layers in the maintenance of undifferentiated human embryonic stem cells (hESCs) growth is not as high as that of mouse feeder cells due to the lower level of secretion of activin A^21^. Although there are numerous chemically defined and xeno-free media such as mTeSR and StemPro conducive to the production of hiPSCs, the inclusion of human serum albumin and human sourced matrix proteins makes those conditions prohibitively expensive, impractical for routine use, and not truly completely defined, which limits their use in large-scale amplification of hiPSCs^22,23^. Thus, the feeder-based system remains an important method of hiPSCs propagation.

Currently, feeder cells are mitotically inactivated either by gamma irradiation^24–30^ or MMC^3,4,11,31–34^. Gamma irradiation can treat more cells than MMC at one time, but the γ-ray radiation source of Cobalt-60 is rare and costly. The affordability, flexibility, and convenience of MMC make it a good routine protocol to prepare feeder cells. For the feeder-based culture system, MEFs of CF-1 strain mice characteristically exhibit active proliferation, high-density dependence, and being aging-prone at low-density, and are still the most common feeder source for hiPSCs cultures.

In the conventional method (CM) for feeder cells preparation^35^, CF-1 MEFs of 80–90% confluence were inactivated and used as feeder cells to maintain hiPSCs or for the production of conditioned medium. However, low yield with high costs need to be optimized as individual dishes or flasks accommodate limited numbers of cells in CM. Failure to fully inactivate MEFs in stratified growth by MMC is another problem. At low density, however, MEFs are aging-prone and their supportive capacities for iPSCs are compromised. Hence, MMC processing time is inflexible. Therefore, it is necessary to find new approaches that not only can be used for the production of feeder cells on a large scale in a short time, but also can ensure that MEF proliferation is sufficiently inhibited. To this end, we recently established a suspension-adhesion method (SAM) and a three-dimensional (3D) suspension method (3DSM) by optimization of CM. These new methods for feeder preparation will promote the advances and applications of induced pluripotent stem cell technology.

## Materials and Methods

### Ethics statement

All methods were carried out in accordance with relevant guidelines and regulations of the Ethics Committee of the Norman Bethune College of Medicine, Jilin University. All experimental protocols were approved by the Ethics Committee of the Norman Bethune College of Medicine, Jilin University. Informed consent was obtained from all subjects. Animal experiments were performed in accordance with a protocol approved by Jilin University School of Medicine Animal Care and Use Committee [Animals use license: SYXK (Jilin) 2013-0005]. All mice were housed in a sterile environment and could access food and water commodiously as outlined in the institutional guidelines.

### Cell culture

CF-1 mouse embryonic fibroblasts (MEFs) were derived from day-12.5 embryo pools of CF-1 strain mice. The cells were cultured in MEF medium [DMEM supplemented with 10% FBS, 1% non-essential amino acids, 1 mM L-glutamine (all from Gibco, Invitrogen, USA)] and maintained at 100% confluence. hHF-MSC-derived iPSCs were cultured as described previously^5^. hHF-MSC-derived iPSCs were maintained on mitotically inactivated CF-1 MEFs in hESCs culture medium (80% DMEM/F12 supplemented with 20% KSR, 1% non-essential amino acids, 1 mM L-glutamine, 4 ng/ml human bFGF, 0.1 mM β-mercaptoethanol) (all from Invitrogen, USA). The hHF-MSC-derived iPSCs were split with 1 mg/ml collagenase type IV (Invitrogen, USA) for 30 min at 37 °C, at a ratio of 1:5 every 6–7 days. Conditioned medium was collected as described^36^. All manipulations and cultivations were performed in a good manufacturing practice (GMP)-compliant facility.

### Preparation of feeder cells with conventional method

CF-1 MEFs of passage 3 (P3) at 80–90% confluence were inactivated with 10 μg/ml of MMC (Hisun Pharmaceutical Company, China) for 0, 0.5, 1.0, 1.5, and 2.0 h at 37 °C. After the incubations, the cells were washed with PBS 6 times, trypsinized, centrifuged at 180 × *g* for 5 min, and re-suspended in MEF medium. Cells were counted and frozen for later use.

### Preparation of feeder cells with suspension-adhesion method

**Figure 1:**
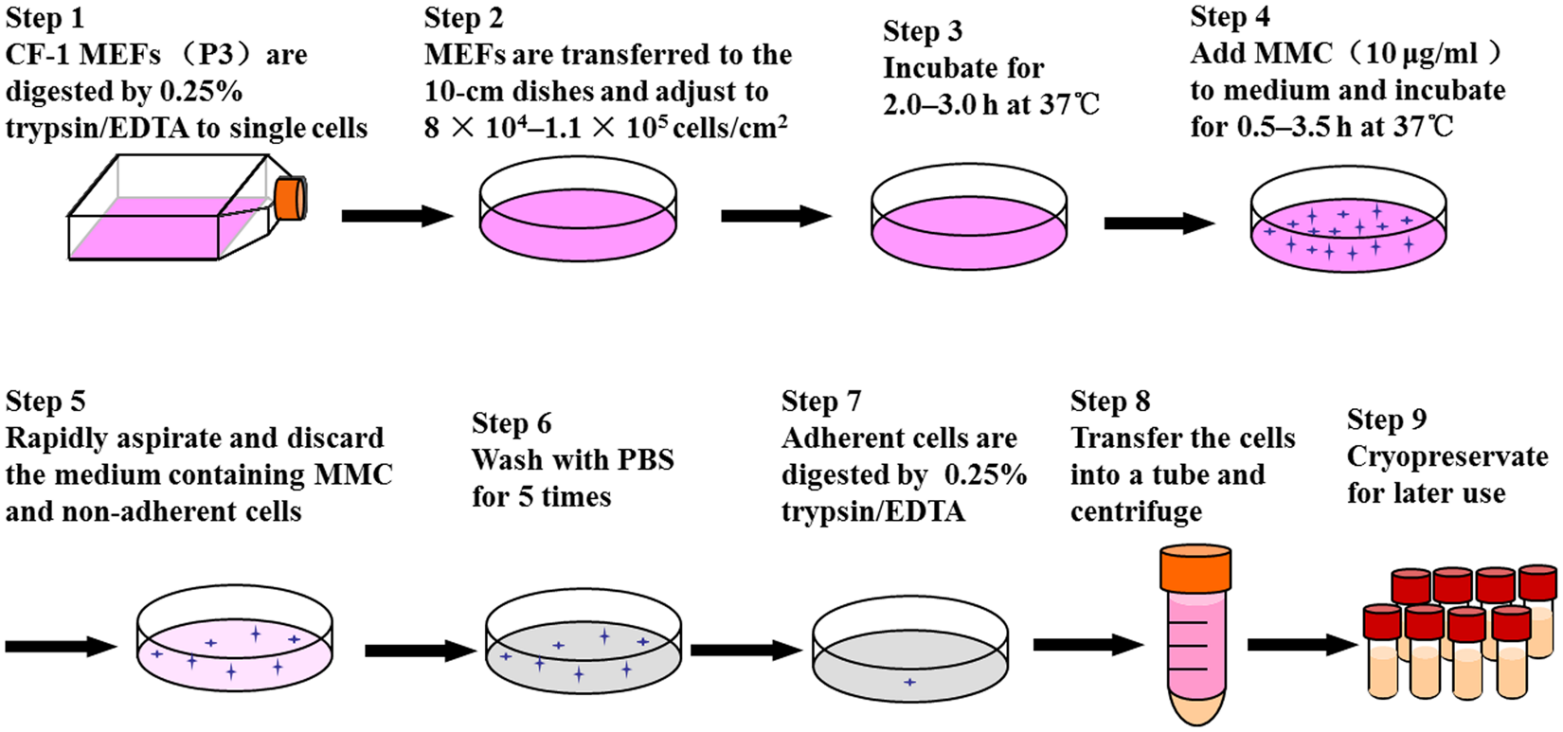
The scheme illustrates the SAM protocol for the preparation of feeder cells.

We prepared feeder cells by SAM according to Fig. 1. Briefly, CF-1 MEFs of P3 were cultured for four days, digested to single cells with 0.25% trypsin/EDTA (Dalian Meilun Biotech Co., Ltd, China), and collected in 50 ml-centrifuge tubes. The cells were seeded at 8 × 10^4^–1.1 × 10^5^ cells/cm^2^ in 10 cm-dishes. MMC (10 μg/ml) was added after 2.0–3.0 h at 37 °C. Medium containing MMC was discarded 0.5–3.5 h post-treatment. The cells were then washed with PBS 6 times, trypsinized, centrifuged at 180 × *g* for 5 min, and resuspended in MEF medium. Cells were counted and frozen for later use.

### Preparation of feeder cells with three-dimensional (3D) suspension method

After connecting the CELLSPIN System (5–75RPM, CELLSPIN System with glass-ball stirring pendulum, Integra Bio-Sciences, Switzerland) to the incubator, spinner flasks were sterilized by autoclaving.

**Figure 2:**
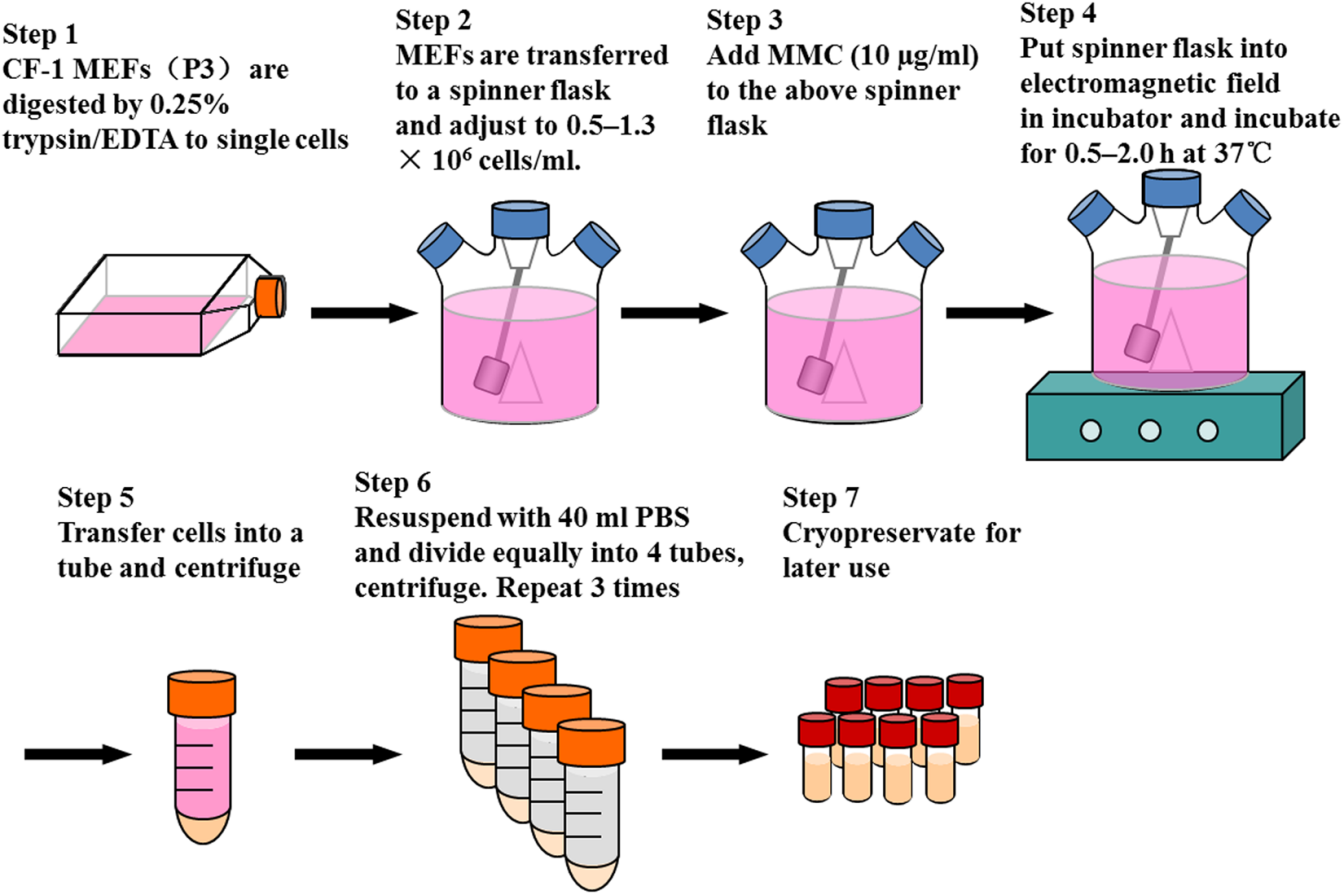
The scheme illustrates the 3DSM protocol for the preparation of feeder cells.

We prepared feeder cells by 3DSM according to Fig. 2. Briefly, CF-1 MEFs of P3 growing for four days were digested to single cells by 0.25% trypsin/EDTA, and collected into a 50 ml-centrifuge tube. Cells were transferred to spinner flasks with glass ball pendulum, which accommodate 25–1000 ml of volume, at a density of 0.5–1.3 × 10^6^ cells/ml. MMC were added at 10 µg/ml. After incubation for 0.5, 1.0, 1.5, and 2.0 h at 37 °C, the cells were centrifuged at 180 × *g* for 5 min, washed with PBS 3 times, resuspended in MEF medium, counted, and cryopreserved for later use.

### Cell count

The feeder cells of CM, SAM and 3DSM were plated at 1 × 10^5^ cells per well in 24-well plates and counted using trypan blue staining solution(Dalian Meilun Biotech Co., Ltd, China) on days 1, 3, 5, and 7.

### 5-ethynyl-2-deoxyuridine (EdU) assay

The inhibitory effect of MMC on the proliferation of MEFs was measured using an EdU assay kit (Ribobio, Guangzhou, China) according to the manufacturer’s instructions. Briefly, inactivated MEFs were cultured in triplicate at 2 × 10^4^ cells per well in 24-well plates. The cells were exposed to 50 μM EdU for 8–24 h at 37 °C. The cells were fixed with 4% formaldehyde for 15 min at room temperature and treated with 0.5% Triton X-100 for 20 min at room temperature for permeabilization. After 3× washes with PBS, the cells were treated with 100 μM 1 × Apollo^R^ reaction cocktail for 30 min. Subsequently, cells were stained with 200 μl/well of Hoechst 33342 (5 μg/mL) for 30 min and visualized under a fluorescent microscope (Olympus, Japan). The number of EdU-positive cells was counted using open-source digitizing software (ImageJ 2.0.0, Wayne Rasband, National Institutes of Health, Bethesda, MD). The percentage of EdU-labeled cells was calculated as follows: EdU-positive cell number/Hochest-positive cell number.

### Comparison of the direct adhesion rates (DAR) of inactivated MEFs prepared by SAM, 3DSM with CM

Inactivated MEFs freshly prepared with CM, SAM and 3DSM were plated at 1 × 10^6^ cells/T25-flask. Non-adherent cells were collected and counted on the second day. Direct adhesion rates were then evaluated.

### Comparison of the recovery rates (RR) of inactivated MEFs prepared by SAM, 3DSM with CM

The frozen MMC-inactivated MEFs of CM, SAM and 3DSM in T25-flasks were resuscitated on days 1, 7, 21, 28 respectively after cryopreservation and the non-adherent cells were collected and counted the following day to analysis the recovery rates of MMC-inactivated MEFs.

### Comparison of the survival time (ST) of inactivated MEFs prepared by SAM, 3DSM with CM

Frozen MMC-inactivated MEFs of CM, SAM and 3DSM were resuscitated and cultured at the same density. Replace the media every three days and observe cell survival time and status.

### Cell cycle assay

Cell cycle *assay* was determined by flow cytometry (BD, USA). Briefly, 1 × 10^6^ inactivated and cryopreserved MEFs of CM, SAM, and 3DSM were plated in T25 flasks. The cells were harvested and fixed in 70% ice-cold ethanol for 24 h, then stained with propidium iodide (PI). The different cell cycle phases were analyzed using a FACS Calibur instrument.

### Trace levels of MMC assayed by HPLC-MS/MS

High performance liquid chromatography-Mass Spectrometry/Mass Spectrometry (HPLC-MS/MS) was performed as described^37^ with modifications. Briefly, a set of MMC calibration solutions with different concentrations were made from a standard stock solution of 1 mg/ml MMC by dilution with methanol: water (1:1, v/v) solution. Aliquots (400–500 μl) were analyzed by HPLC-MS/MS to construct a standard curve. All chomatographic experiments were carried out at room temperature using HPLC (Agilent technologies, USA) equipment. MS/MS analysis was performed using LCQ Deca XP ^plus^ equipment (Agilent technologies, USA) with an ESI source in the positive ion mode. The daughter ions with m/z 274 for MMC were monitored via an ion trap mass analyzer.

### hHF-MSC-derived iPSCs pluripotency marker assay

The hHF-MSC-derived iPSCs pluripotency markers were assayed using a Fluorescent Human ES/iPS Cell Characterization Kit from EMD Millipore (Millipore, USA) according to the manufacturer’s instructions.

### Embryoid body-mediated differentiation of hHF-MSC-derived iPSCs

hHF-MSC-derived iPSCs were harvested by collagenase type IV. After settling, the supernatant was aspirated and the MEF medium was replaced to remove the MEF. hHF-MSC-derived iPSCs were transferred to petri dishes in the MEF medium. After an 8 days floating culture, embryoid bodies were transferred to gelatin-coated plates and were then incubated for another 16 days. After the incubation, the cells were fixed with 4% paraformaldehyde in PBS and then incubated in PBS containing 5% normal goat serum (Maixin Biotech, Fuzhou, China), 1% bovine serum albumin (BSA, Biotopped, China), and 0.2% Triton X-100. The primary antibodies were as follows: anti-alpha smooth muscle actin (α-SMA, R&D, USA), anti-alpha fetoprotein polyclonal antibody (AFP, R&D, USA), and anti-Nestin (R&D, USA). vimentin(Cell Signaling, USA), desmin (Cell Signaling, USA), βIII-tubulin (Cell Signaling, USA). The secondary antibodies were Alexa 555-labeled anti-mouse IgG (1:1000, Cell Signaling, USA). Nuclei were stained with 1 mg/ml Hoechst 33342 (Invitrogen, USA).

### Karyotype analysis

Karyotype of the hHF-MSC-derived iPSCs analysis was evaluated and performed at the Reproductive Center of the First Hospital of Jilin University using standard protocols for high-resolution G-banding.

### Teratoma formation

The hHF-MSC-derived iPSCs were injected intramuscularly into non-obese diabetic/severe combined immune deficient (NOD/SCID) mice in DMEM containing 10% FBS (3 × 10^6^ cells per site). After 12 weeks, teratomas were retrieved from the injection site, dissected, and fixed with 10% formaldehyde in PBS. Paraffin embedded tissue sections were then prepared and analyzed with Hematoxylin and Eosin (H&E) staining.

### Statistical analysis

The significance level was determined by Student’s t test and ANOVA. All quantitative data presented were the Means ± SD from at least three independent experiments.

## Results

### Preparation of feeder cells

**Figure 3:**
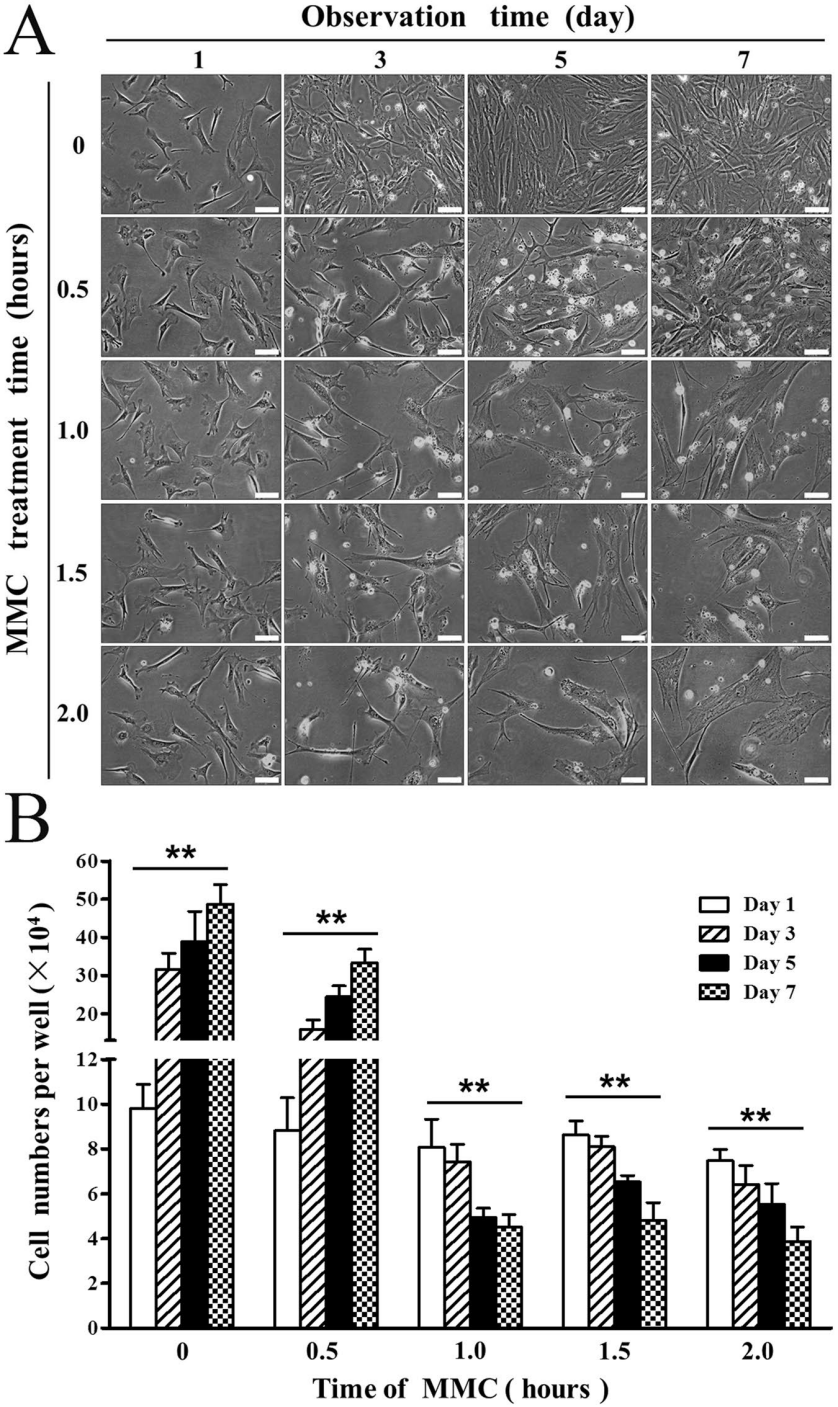
Inhibition of MEF growth by MMC in CM. (**A**) Phase contrast microscopy images of MEFs treated with 10 µg/ml MMC for 0–2.0 h in CM and observed on days 1, 3, 5 and 7. Bars = 40 μm. (**B**) Cell counts of inactivated MEFs in each well of 24-well plate (Student’s t test and ANOVA; **P < 0.01 versus each group of days 1, 3, 5 and 7).

We prepared feeder cells by conventional method (CM) as previous reports^3,4,35^. Briefly, we harvested MEFs from embryonic day 12.5 embryo pools of CF-1 mice. The primary cells contained a variety of different types of cells. CF-1 MEFs became more homogeneous at passage 3 (P3) and were used as feeders. MEFs were treated with 10 μg/ml MMC for 0, 0.5, 1.0, 1.5, or 2.0 h respectively. Then, the culture media were replaced with MEF media and cell numbers were counted on days 1, 3, 5, and 7. As shown in Fig. 3, phase contrast microscopy images and cell counts demonstrated that the number of cells significantly increased (*P* < 0.01) in the culture on days 1, 3, 5, and 7 after being treated with 10 µg/ml MMC for 0.5 h by CM, indicating that MMC treatment for 0.5 h failed to inhibit MEF proliferation in CM. However, the number of cells significantly decreased in CM when using MMC for 1.0, 1.5, and 2.0 h, and statistically significant differences were found between days 1, 3, 5, and 7 (*P* < 0.01). It is worth mentioning that some of the cells were unhealthy in the MMC 1.0–2.0 h groups and died in three days.

**Figure 4:**
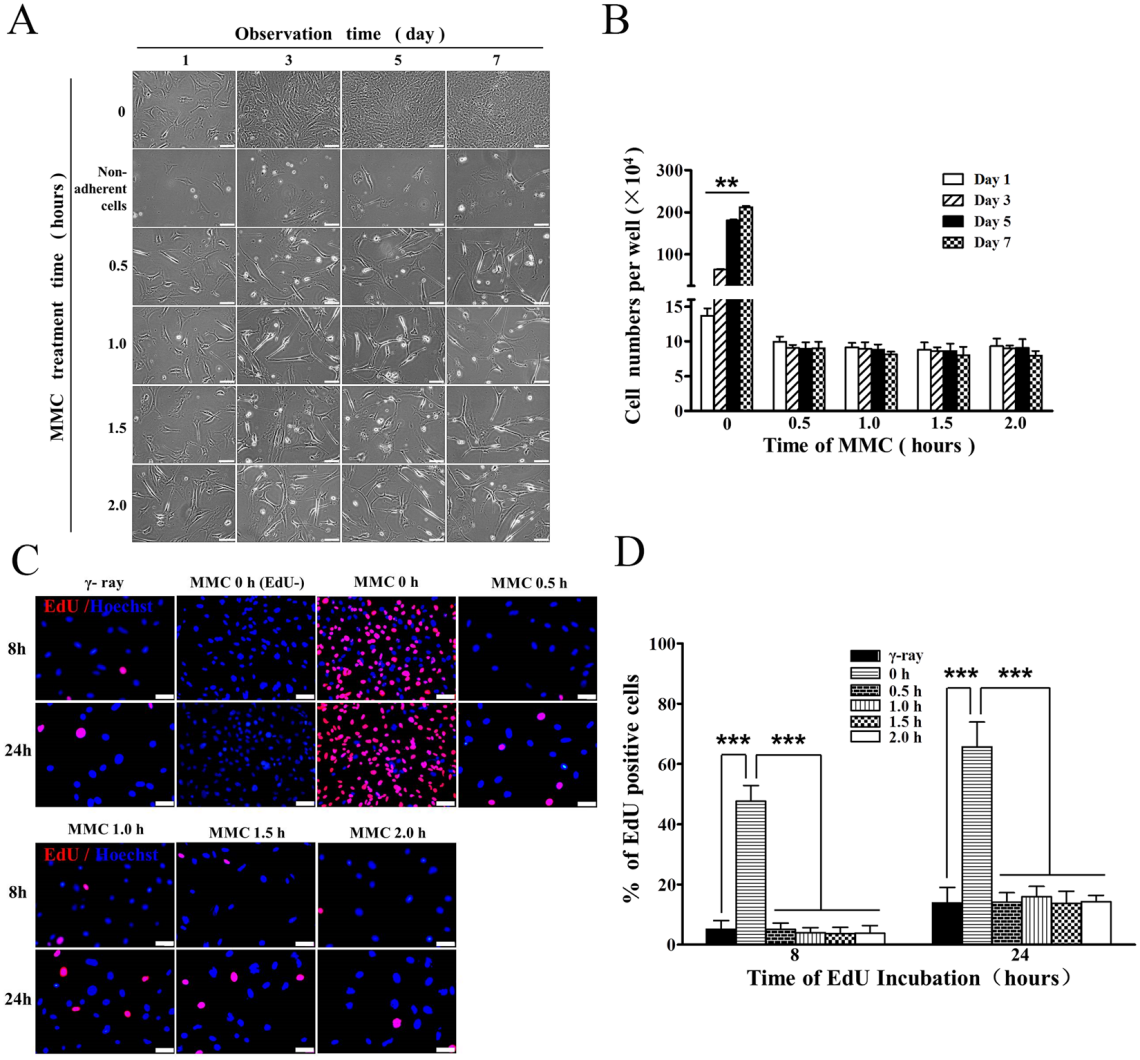
Inhibition of MEF growth by MMC in SAM. (**A**) Phase contrast microscopy images of MEFs treated with 10 µg/ml MMC for 0–2.0 h in SAM and observed on days 1, 3, 5 and 7. Bars = 40 μm. (**B**) Cell counts of inactivated MEFs in each well of 24-well plate (Student’s t test and ANOVA; **P < 0.01 versus each group of days 1, 3, 5 and 7). (**C**) EdU-positive cells and representative images of fields show red-colored proliferation cells. Hoechst 33342 staining was performed to detect nuclear localization. Bars = 40 μm. (**D**) The number of EdU-positive cells were counted using ImageJ (Student’s t test; ***P < 0.001).

To optimize CM, we first prepared feeder cells according to Fig. 1. Several reports suggested that 10 μg/ml MMC is sufficient to inhibit proliferation of MEFs^21,36,38–45^. To identify the optimal time of inhibition in suspension-adhesion method (SAM), we performed a time course experiment, exposing the MEFs to 10 µg/ml MMC for 0–6.0 h. Phase contrast microscopy images and cell counts (Fig. 4A,B and Fig. S1) showed that MEFs statistically significant increased in the cell numbers on days 1, 3, 5, and 7 without MMC treatment (*P* < 0.01). MEFs exposed to MMC for 0.5–4.0 h did not change in number over 7 days. The number of cells did not perceptibly increase or decrease on days 1, 3, 5, or 7 for cells treated with MMC for 0.5, 1.0, 1.5, 2.0, 2.5, 3.0, 3.5, or 4.0 h, and no statistically significant differences were found in cell numbers on days 1, 3, 5, or 7. However, the number of cells decreased on days 1, 3, 5, and 7 for MEFs treated with MMC for 4.5, 5.0, and 6.0 h (*P* < 0.05) probably due to cell death. Take together, our data suggest that treatment with 10 µg/ml MMC for 0.5–4.0 h is sufficient to suppress the proliferation of MEFs but maintains acceptable viability of feeder cells. Thus, we prepared feeder cells with SAM using 10 µg/ml MMC for 0.5–4.0 h.

To further validate the suppressive effect of MMC on the proliferation of MEFs, we labeled the cells with EdU and determined the cell proliferation rate after MMC treatment (Fig. 4C,D and Table S1). There were statistically significant differences between untreated cells and those treated with MMC for each time point between 0.5–3.5 h and gamma ray (*P* < 0.001). However, there was no significant difference between the gamma ray group and cells treated with MMC for 0.5–3.5 h (data of 2.0–3.5 h are not shown). Together, these results further confirmed that 10 μg/ml MMC for 0.5–3.5 h was sufficient to inhibit MEF proliferation.

**Figure 5:**
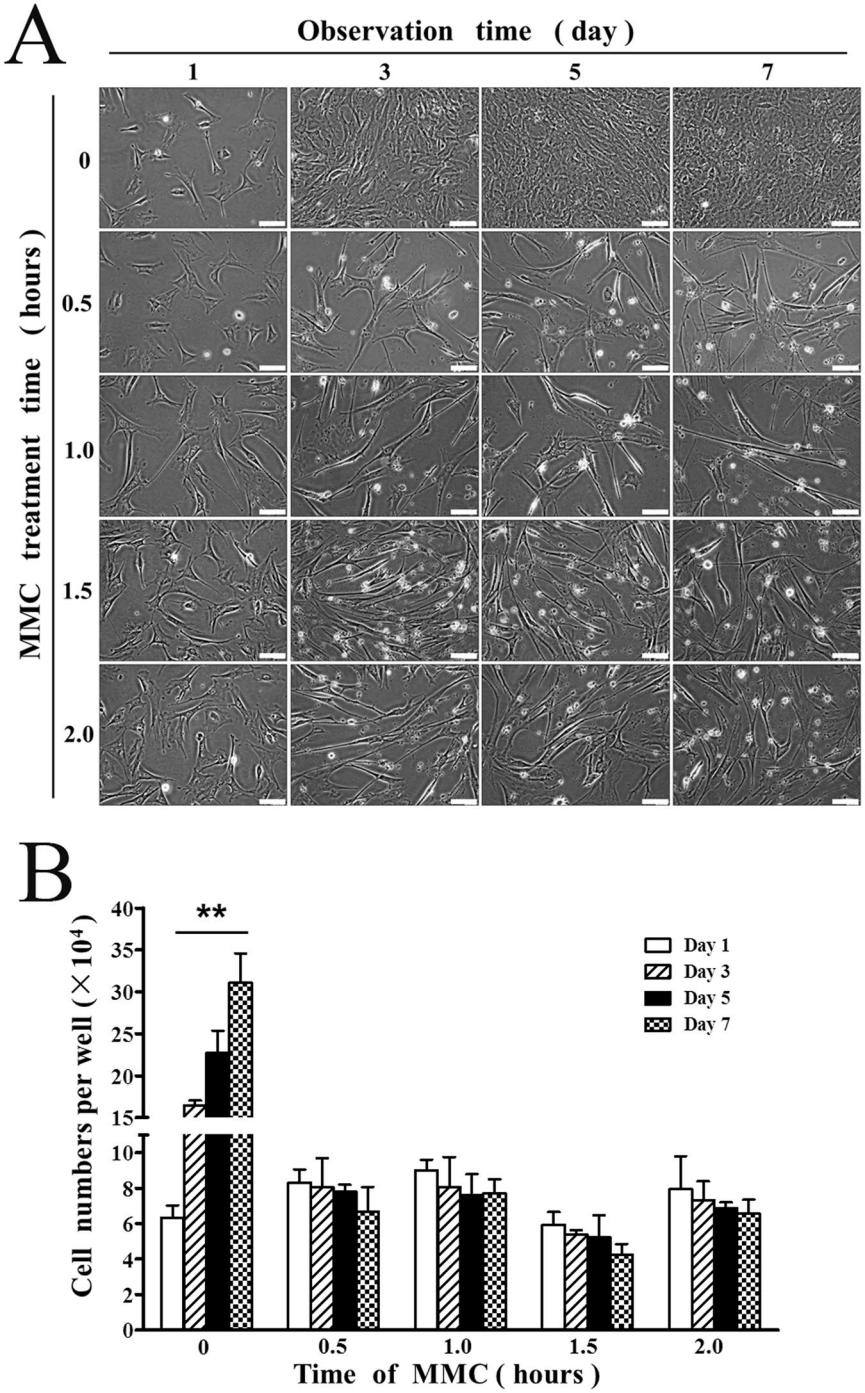
Inhibition of MEF growth by MMC in 3DSM. (**A**) Phase contrast microscopy images of MEFs treated with 10 µg/ml of MMC for 0–2.0 h in 3DSM and observed on days 1, 3, 5 and 7. Bars = 40 μm. (**B**) Cell counts of inactivated MEFs in each well of 24-well plate (Student’s t test and ANOVA; **P < 0.01 versus each group of days 1, 3, 5 and 7).

Both the SAM and CM require MEFs to attach to the flasks/dishes, which limits the processing efficiency of the feeder cells. To increase the processing efficiency and reduce costs (Table 1), we used the three-dimensional (3D) suspension method (3DSM) to re-optimize the SAM for the preparation of feeder cells (Fig. 2). MEFs were treated with 10 μg/ml MMC for 0–2.0 h. Then, we observed and counted cell numbers on days 1, 3, 5, 7. Phase contrast microscopy images and cell counts (Fig. 5A,B) demonstrated that without MMC the number of MEFs significantly increased on days 1, 3, 5, and 7 in 3DSM (*P* < 0.01). The number of cells did not change on days 1, 3, 5, or 7 after treatment with MMC for 0.5–2.0 h.

**Table 1:** Comparison of CM, SAM, and 3DSM.

Method	CM	SAM	3DSM	
Dish/Bottle size	10 cm-dish	10 cm-dish	60 ml in 1 × 100 ml SF	800 ml in 1 × 1000 ml SF
Yield ( × 10^6^)	3.5–5	4.5–6	> 70	> 900
Cost	High	High	Low	Low
SPT (hours)	1.0	0.5	0.5	0.5
Manipulation	Inflexible	Flexible	Flexible	Flexible
Workload	Heavy	Heavy	Light	Light
DAR	70–85%	96%	90–96%	90–96%
RR	70–73%	85–92%	83–90%	83–90%
ST (days)	<7	>7	>7	> 7

SF, spinner flask; SPT, shortest processing time; DAR, direct adhesion rate; RR, recovery rate; ST, survival time.

### Comparison SAM, 3DSM with CM

To determine the quality of feeder cells prepared by SAM and 3DSM, we compared the shortest processing time (SPT) of inhibition MEF proliferation, the direct adherent rates (DAR), recovery rates (RR) and survival time (ST) of MEFs harvested from SAM, 3DSM and CM.

The aforementioned data showed that MMC for 0.5 h was sufficient to inhibit the proliferation of MEFs in SAM and 3DSM (Figs 4A,B and 5A,B). But MMC for 0.5 h failed to inhibit MEF proliferation in CM (Fig. 3A, B). Thus, SAM and 3DSM were more efficient than CM in terms of the suppression of MEF proliferation. In addition, the number of MEFs in SAM and 3DSM did not perceptibly increase or decrease on days 1, 3, 5, or 7 for those treated with MMC for 0.5–2.0 h. But some of the MEFs treated with MMC for 1.0–2.0 h in CM were unhealthy and died in three days. Thus, it seems that the feeder cells prepared in SAM and 3DSM may be higher quality as those prepared in CM.

**Figure 6:**
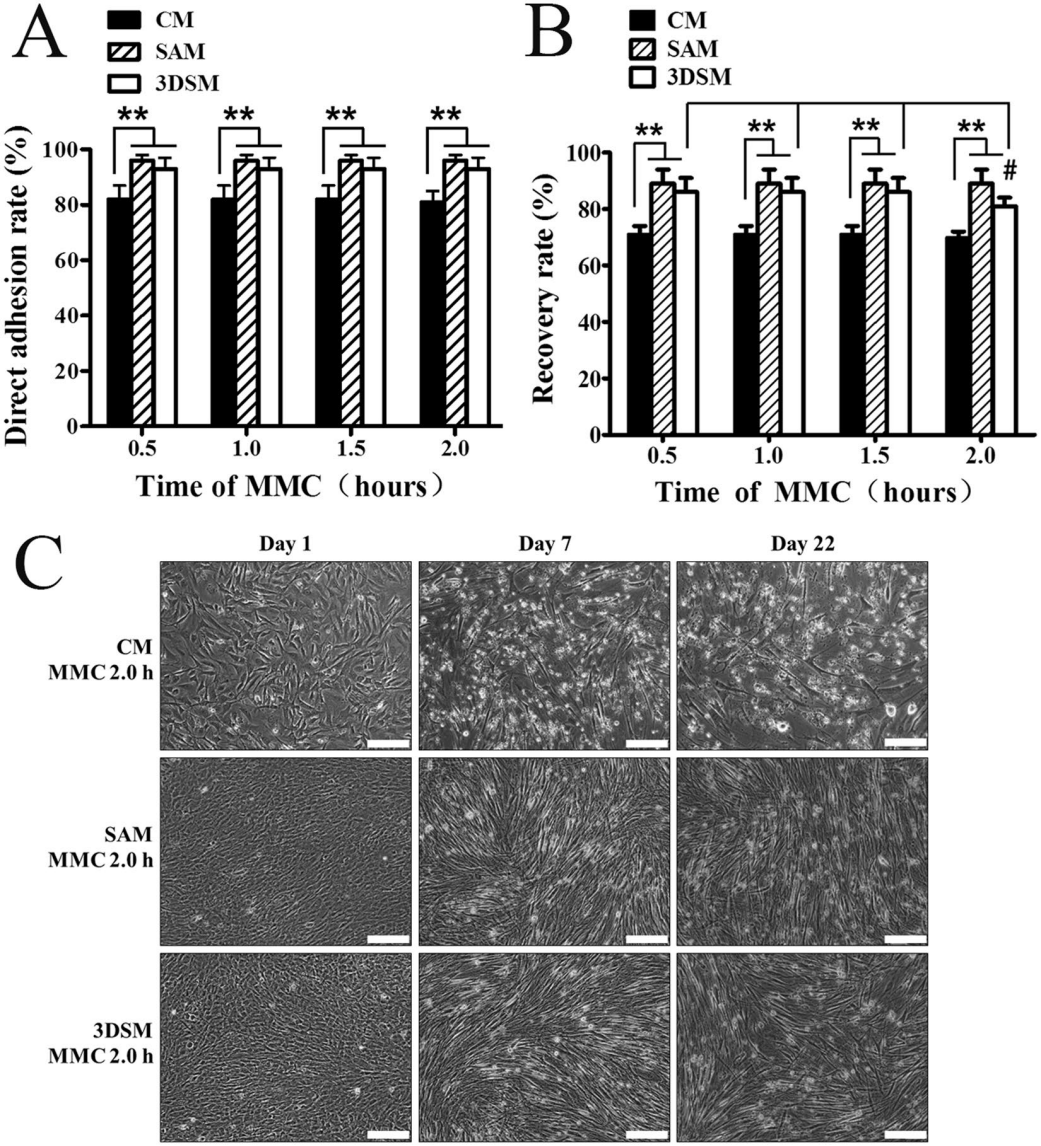
Comparison of feeder cells of CM with SAM and 3DSM. (**A**) The direct adhesion rates of inactivated MEFs treated with MMC for 0.5, 1.0, 1.5, and 2.0 h in CM, SAM and 3DSM (Student’s t test and ANOVA; **P < 0.01). (**B**) The recovery rate of inactivated MEFs, which were treated with MMC for 0.5, 1.0, 1.5, and 2.0 h in CM, SAM and 3DSM (Student’s t test; **P < 0.01, ^#^ *P < *0.05 versus the 2.0 h with 0.5, 1.0 and 1.5 h). (**C**) Long-term culture of inactivated MEFs treated with MMC for 2.0 h in CM, SAM and 3DSM. Bars = 100 μm.

Next, we compared the DAR of inactivated MEFs treated with MMC for 0.5, 1.0, 1.5, and 2.0 h in CM with SAM and 3DSM (Fig. 6A). The DAR of inactivated MEFs treated with MMC for 0.5–2.0 h in SAM could reach 96% ± 2% and 3DSM could also reach 93% ± 4%, however, the rate in CM was 82% ± 5%. Our data indicated that the DAR of MEFs in SAM and 3DSM were higher than that in CM.

Similarly, the RR of inactivated MEFs treated with MMC for 0.5–2.0 h in SAM was up to 89% ± 5% and 3DSM was 86% ± 3%, whereas the rate in CM was 71% ± 3% (Fig. 6B), indicating a better recovery for feeder cells prepared by SAM and 3DSM.

Notably, the RR of 3DSM-feeder cells treated with MMC for 0.5, 1.0, or 1.5 h were higher (86% ± 5%) than that for 2.0 h (81% ± 3%) (Fig. 6B). These findings demonstrate that MEFs in 3DSM inactivated with MMC for 0.5, 1.0, or 1.5 h duplicate the characteristics of SAM. Thus, we prepared feeder cells by 3DSM using treatment with MMC for 0.5–1.5 h.

We then cultured the MEFs inactivated with MMC for 2.0 h by CM, SAM and 3DSM at a high density (Fig. 6C). The survival of feeder cells from CM was markedly lower than that of feeder cells from SAM and 3DSM on day 1. This may be in part due to the low recovery in CM-prepared feeder cells. Indeed, we observed a large number of dead cells in CM-prepared feeder cells on day 7. In contrast, SAM/3DSM-feeder cells at high density maintain viability for up to 22 days.

Thus, our data suggest that SAM and 3DSM were more efficient than CM in inhibiting cell proliferation. SAM/3DSM-derived feeder cells can better maintain long-spindle-cell growth, and exhibit an improved DAR, RR, and ST compared to those derived using the CM. Therefore, these data further support our conclusion that the qualities of feeder cells prepared in SAM and 3DSM are better than that in CM. Furthermore, SAM and 3DSM may be able to address the issues important for the application of feeder cells for hiPSCs culture, such as failure of full mitotic inactivation, poor quality of the feeder cells, and over-growth of the MEFs.

### Cell cycle analysis of feeder cells prepared by CM, SAM, or 3DSM

**Figure 7:**
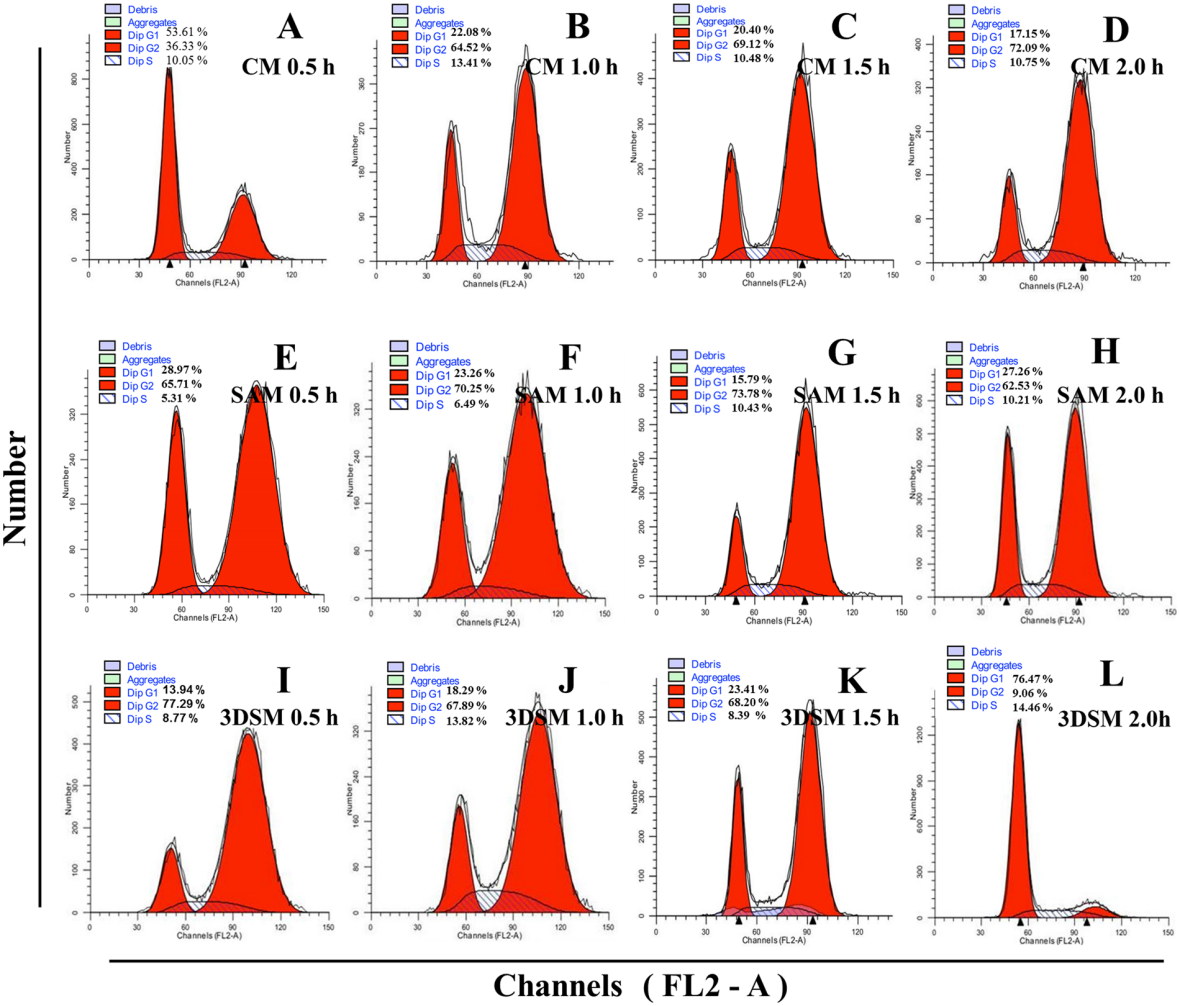
Cell cycle analysis of feeder cells prepared by CM, SAM and 3DSM. Feeder cells prepared by CM (**A**–**D**), SAM (**E**–**H**), and 3DSM (**I**–**K**) with the treatment of MMC, and without MMC treatment (**L**) were labeled with PI and analyzed with flow cytometry for the cell cycle distribution.

To determine the cell cycle distribution of the inactivated MEFs prepared from CM, SAM, and 3DSM, we cultured the cells for 1 day, labeled them with PI, and analyzed them using flow cytometry. As shown in Fig. 7, untreated MEFs exhibited a typical cell cycle distribution with a relatively high level of G1 (76.47%, Fig. 7L). Treatment with MMC led to arrest in G2 (Fig. 7A–K and Fig. S2), consistent with previous report for the action of MMC^46^. However, there is no statistically significant difference in the distribution of cells prepared with CM (Fig. 7B–D), SAM (Fig. 7E–H), and 3DSM (Fig. 7I–K). In addition, there is no significant cell apoptosis in the cultures as judged by the sub-G1/G0 distributions of the cells in the flow cytometry analysis.

Interestingly, inhibition was not complete with MMC for 0.5 h in CM, which cell cycle distribution of MEFs was between group MMC 0 h and group proliferation was completely inhibited (Fig. 7A). This is also consistent with our previous finding that MMC 0.5 h by CM is not sufficient to inhibit cell proliferation.

### Trace levels of MMC detected by HPLC–MS/MS

In long-term hiPSCs culture, abnormal karyotypes occurred after 10 passages when the trace MMC concentration was higher than 0.02 ng/ml^37^.

**Figure 8:**
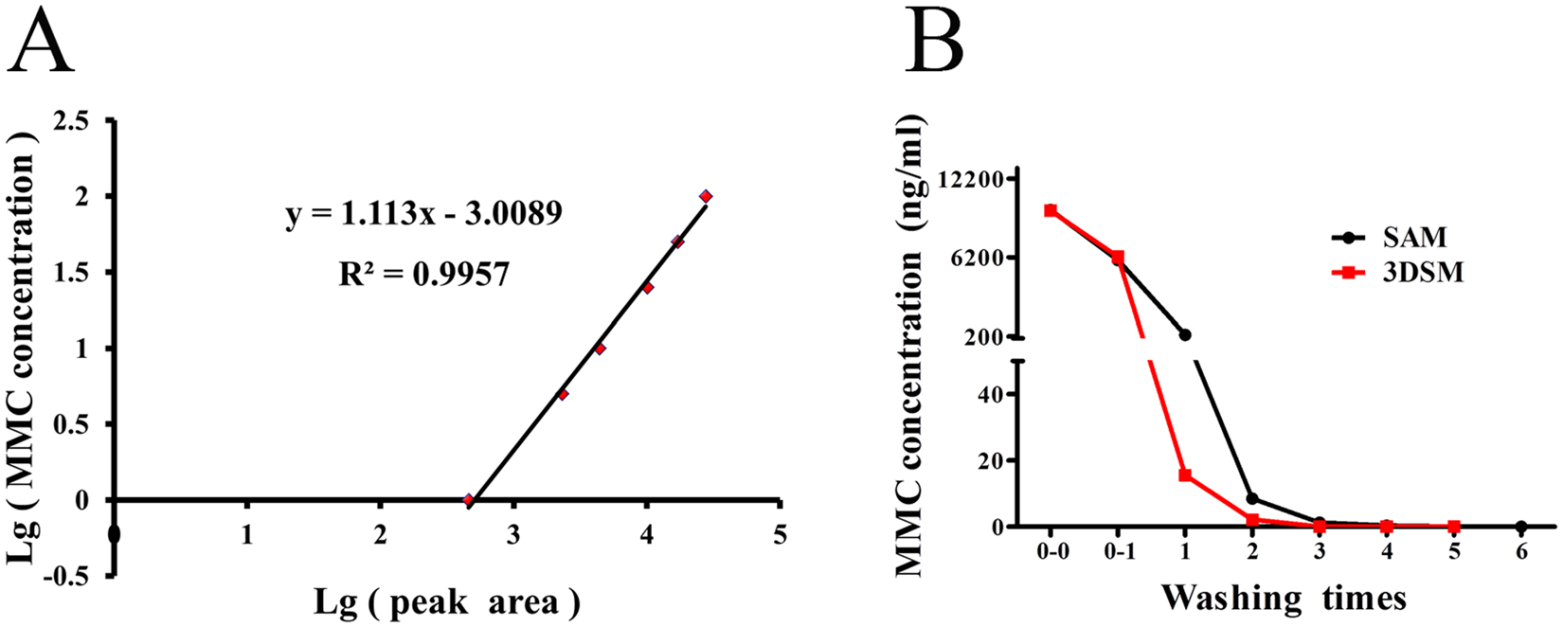
Detection of residual MMC at each washing step. (**A**) MMC standard curve made by HPLC-MS/MS. Standard stock solution (1 mg/ml) was diluted into six MMC standard solutions ranging from 1 to 100 ng/ml. (**B**) Trace levels of MMC at each washing time detected by HPLC–MS/MS.

To remove the MMC as much as possible, typically, we washed 6 times and 5 times with PBS in the preparation process of SAM and 3DSM respectively. To identify whether MMC remains in hHF-MSC-iPSC culture systems after several washing steps and to identify the optimal washing times, we analyzed residual MMC levels using HPLC–MS/MS. A series of MMC standard solutions of 100, 50, 25, 10, 5, 1 ng/ml were prepared, a standard curve was performed (Fig. 8A).

We found that 9805.52 ± 302.08 ng/ml and 9752.32 ± 267.43 ng/ml MMC were the initial concentration of MMC used for inactivating MEFs and concentration convert to 5999.56 ± 632.08 ng/ml and 6234.56 ± 572.38 ng/ml after treatment in SAM and 3DSM separately (Fig. 8B). MMC concentration decreased rapidly with increasing washing times. At the 4th washing in SAM, the residual MMC concentration in washing PBS was 0.3337 ± 0.06 ng/ml. MMC was undetectable in washing PBS at the 5th and the 6th washing in SAM. At the 2nd washing in 3DSM, the residual MMC concentration in washing PBS was 2.1627 ± 0.16 ng/ml MMC was undetectable afterwards in 3DSM.

MMC was undetectable in hHF-MSC-iPSC medium. Therefore, we believe that wash 5 times in SAM and 3 times in 3DSM are sufficient to remove MMC, and residual MMC in hHF-MSC-iPSC medium is minimal and safe.

### Co-culture hHF-MSC-derived iPSCs with feeder cells prepared by SAM and 3DSM

**Figure 9:**
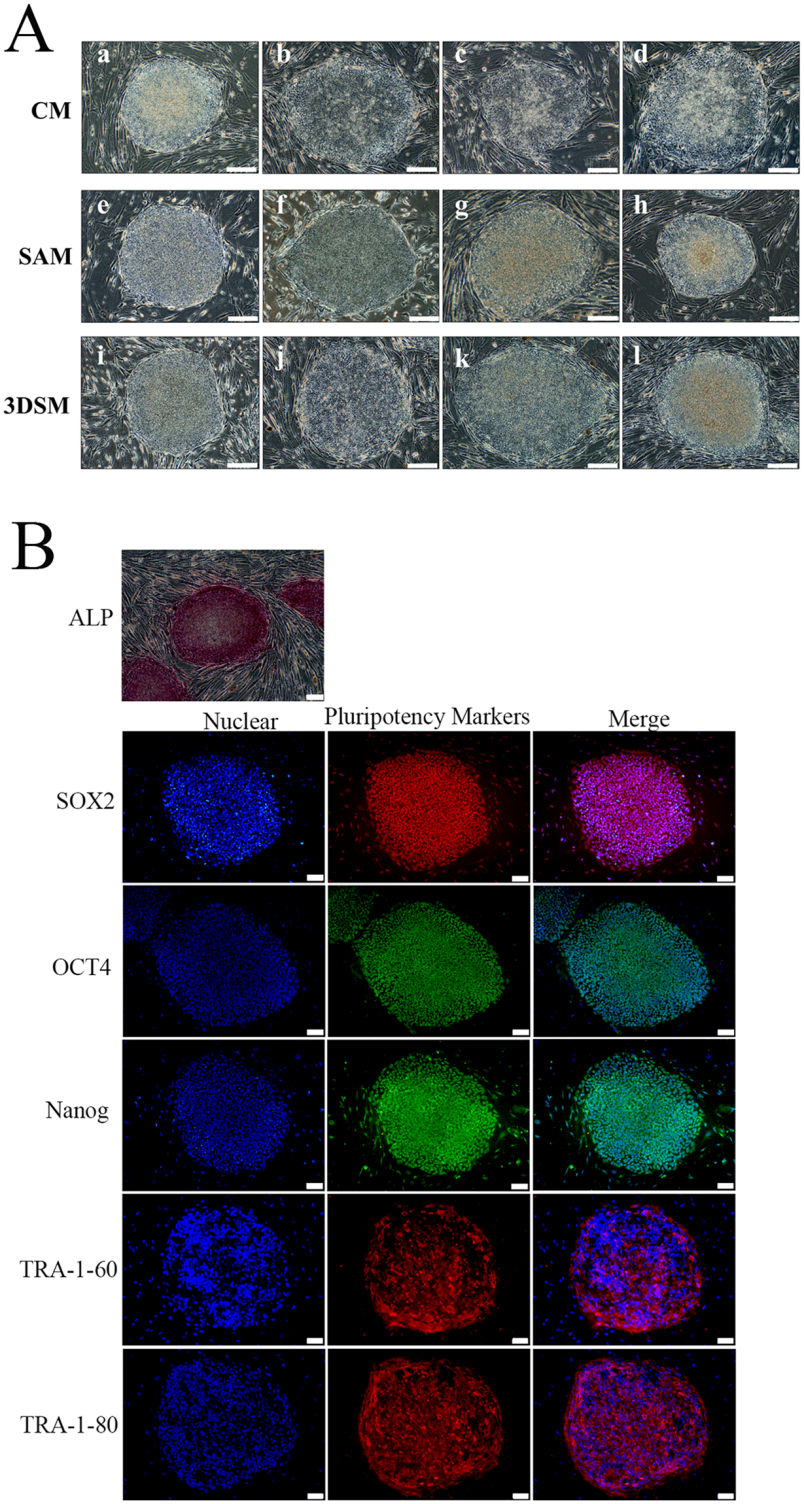
Typical hESCs-like colonies and hESCs pluripotency markers expressed in hHF-MSC-iPSCs. (**A**) hHF-MSC-iPSCs cultured on feeder cells prepared by CM, SAM and 3DSM exhibited typical hESCs-like colonies: Aa. hESCs colonies, X-01; Ab-d. hHF-MSC-derived iPSCs in CM-feeder cells treated with MMC for 1.0, 1.5, and 2.0 h respectively. Ae–h. hHF-MSC-derived iPSCs in SAM-feeder cells treated with MMC for 0.5, 1.0, 1.5, and 2.0 h, respectively; Ai–l. hHF-MSC-derived iPSCs in 3DSM-feeder cells treated with MMC for 0.5, 1.0, 1.5, and 2.0 h respectively. Bar = 100 μm. (**B**) Pluripotency markers expressed by iPSCs: hHF-MSC-derived iPSCs were fixed and stained with anti-bodies against pluripotency makers proteins and stained nuclei with hoechst33342. Bars = 50 μm.

hHF-MSC-derived iPSCs characterized by a high nucleus to cytoplasm ratio and prominent nucleoli (Fig. 9Aa). The hHF-MSC-derived iPSCs may be propagated continuously without losing undifferentiated status for more than 10 passages (>60 days) with CM-feeder cells treated with MMC for 1.0–2.0 h (Fig. 9Ab–d), more than 60 passages (>400 days) with SAM-feeder cells treated with MMC for 0.5–3.5 h (Fig. 9Ae–h), and more than 10 passages (>60 days) with 3DSM-feeder cells treated with MMC for 0.5–2.0 h (Fig. 9Ai–l). The hHF-MSC-derived iPSCs expressed typical hESCs surface markers, including TRA-1-60 and TRA-1-81. The cells also expressed pluripotent markers, including alkaline phosphatase (AP), Oct 4, Sox2, and Nanog (Fig. 9B).

### Pluripotency of hHF-MSC-derived iPSCs: embryoid body-mediated differentiation and teratoma formation of hHF-MSC-derived iPSCs

**Figure 10:**
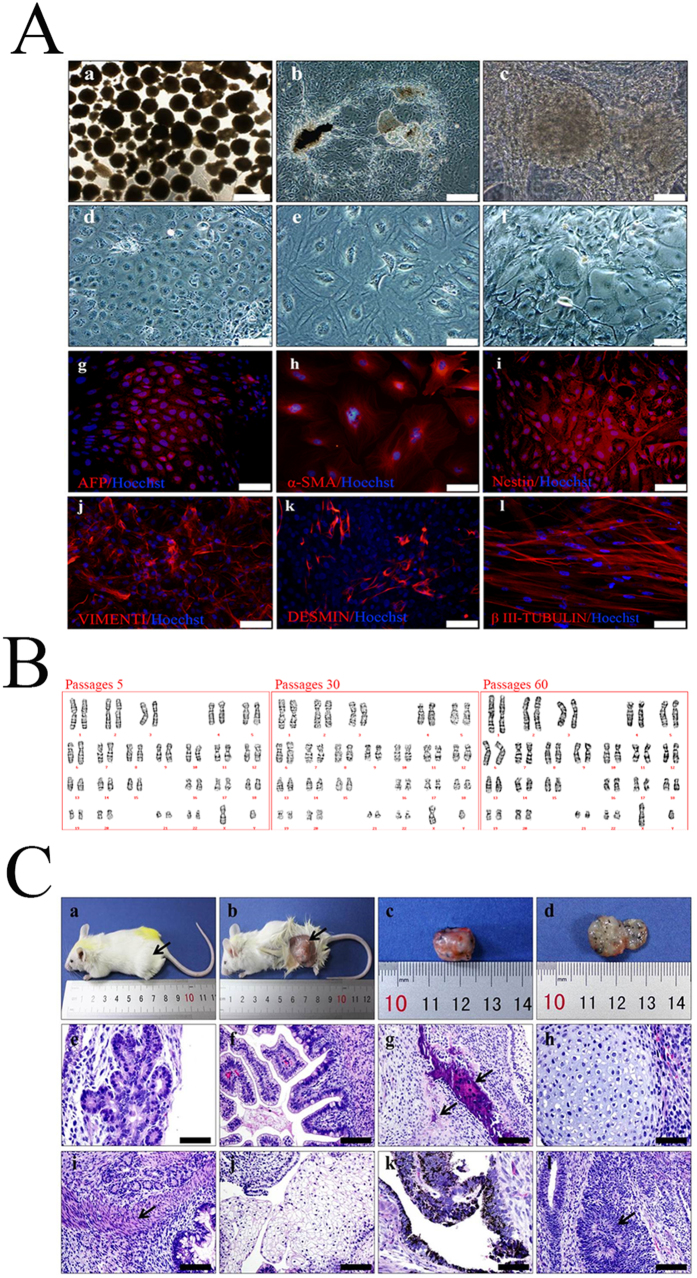
Pluripotency of hHF-MSC-derived iPSCs. (**A**) *In vitro* embryoid body formation and differentiation. Immunostaining confirmed *in vitro* differentiation into all three germ layers. Secondary antibodies were labeled with Alexa 555 (red). A spontaneous contracting cluster developed following differentiation (Ac). Bars = 400 μm (a), 100 μm (b), 600 μm (**c**), 40 μm (d- l). (**B**) Karyotype of hHF-MSC-derived iPSCs. G-band analysis of karyotypes of hHF-MSC-derived iPSCs at passages 5, 30, and 60, respectively. The hHF-MSC-derived iPSCs retained a normal karyotype at passages 30 and 60 compared with passages 5. (**C**) In vivo teratoma formation. Ca–d. Gross observation of teratoma. There was a tumor-like protrusion in outer thighs as indicated by the arrow (Ca, b). Tumor size was 1.3 cm × 1.0 cm × 1.0 cm (Cc). The cleavage plane of teratoma (Cd). Hematoxylin and eosin staining of teratoma derived from hHF-MSC-derived iPSCs revealed that the tumor contained various tissues of the three germ layers, including gut-like epithelial tissues (Ce, endoderm), digestive tract epithelium (Cf, endoderm), bone tissue and osteoid tissue (Cg, mesoderm), cartilage (Ch, mesoderm), smooth muscle tissue (Ci., mesoderm), pavement epithelium (Cj, ectoderm), pigmented epithelium (Ck, ectoderm), neural tissues (Cl, ectoderm). A tumor developed from one injection site. Bars = 50 μm (e, h, k), 100 μm (f, g, i, j, l).

hHF-MSC-derived iPSCs formed embryoid bodies in non-coated plastic dishes (Fig. 10Aa). When cultured in gelatin-coated dishes, the embryoid bodies from hHF-MSC-iPSCs attached to the dish and initiated differentiation (Fig. 10Ab–f). After 24 d, immunocytochemistry detected cells positive for α-fetoprotein (AFP, endoderm, Fig. 10Ag), and vimentin (mesoderm and parietal endoderm, Fig. 10Aj), α-smooth muscle actin (α-SMA, mesoderm, Fig. 10Ah), desmin (mesoderm, Fig. 10Ak), nestin (ectoderm, Fig. 10Ai), βIII-tubulin (ectoderm, Fig. 10Al). The hHF-MSC-derived iPSCs which were subjected to long-term cultivation had a normal karyotype. The hHF-MSC-derived iPSCs retained a normal karyotype at passages 30 and 60 compared with passages 5 (Fig. 10B).

hHF-MSC-iPSCs were injected intramuscularly into NOD/SCID mice. Five weeks after injection, we observed tumor formation. After 12 weeks, tumor size was 1.3 cm × 1.0 cm × 1.0 cm. Histological examination revealed that the tumor contained various tissues of the three germ layers, including gut-like epithelial tissues (endoderm), digestive tract epithelium (endoderm), bone tissue and osteoid tissue (mesoderm), cartilage (mesoderm), smooth muscle tissue (mesoderm), pavement epithelium (ectoderm), pigmented epithelium (ectoderm), neural tissues (ectoderm) (Fig. 10C).

## Discussion

Our data suggest that the qualities of feeder cells prepared in SAM and 3DSM are better uniformity, more standardized than that in CM. SAM and 3DSM could not only increase the processing efficiency of feeder cells but also reduce costs, and lower workload (Table 1). Flexibility and convenience are additional advantages of SAM and 3DSM to prepare feeder cells.

In SAM, MEFs are in single cell suspension and have more accessibility to MMC than CM, therefore are easier to be inhibited by MMC. Only those healthy MEFs that adhere to culture dish/flask bottom are collected. Besides, additional 4-day culture of P3 MEFs increased 300% cell yield. Additionally, no 80%-90% growth restriction for MMC treatment is necessary in SAM or 3DSM.

3DSM has additional advantages over SAM: no subculture is needed, hence less workload; and less culture dishes/flasks are used. In the 3DSM, the MEFs are in suspension during the entire process and access MMC thoroughly, which could improve the efficiency of inhibiting cell proliferation. In addition, since enzymatic digestion is omitted during feeder cell preparation, no damaged cells caused by enzyme digestion exist in our culture system which may also contribute to the high yield of MEFs in 3DSM. According to the density of the treated cells, the yield of a single spinner flask in 3DSM was greater than that achieved using a 10-cm dish, in the same volume of culture medium. Therefore, 3DSM can be used to rapidly produce feeder cells on a large-scale which could be very helpful in mass production of hiPSCs.

hiPSCs were feeder-density dependent^47^. Feeder cells at high density (30,000 cells/cm^2^ and above) may cause rapid depletion of nutrients and oxygen and physically hinder the attachment and growth of hiPSCs. MEFs at low density may result in insufficient levels of extracellular matrix, secreted factors, and intercellular contacts. Newly prepared feeder cells are used, DAR of the cells would affect the feeder cell density. That is, the higher DAR, the higher feeder cell density. Variability in feeder cell density may affect the outcome of hiPSCs culture. Our data showed that the adhesion rates of freshly prepared and frozen feeder cells in SAM and 3DSM were far higher than in CM. Thus, less SAM and 3DSM feeder cells are needed to support hiPSCs.

It usually takes the cells 4–20 days for hHF-MSC-derived iPSCs to revive from cryopreservation and the routine cultures were split every 5–7 days. Our data (Fig. 6C) showed frozen feeder cells treated with 10 μg/ml MMC for 2.0 hours in CM suffered a marked apoptosis within the first three days of culture. In stark contrast, feeder cells prepared in SAM and 3DSM remain healthy for up to 7 days and there is no significant cell death found until day 22. Thus, feeder cells obtained from SAM and 3DSM should be sufficient to support routine culture and recovery of hiPSCs. Moreover, hiPSCs cultured on feeder cells in SAM and 3DSM are less likely to differentiate or are in better condition than those on feeder cells prepared in CM.

The development of reprogramming technology promotes the advance of stem cell biology research. Adult cell reprogramming by ectopic coexpression of transcription factors and gene editing brings new hope to the treatment of many human diseases, especially personalized regenerative medicine treatments. In response to the requirement of rapid and large-scale hiPSCs production, it has become a very active research topic preparing a large number of feeder cells of high-quality. We developed SAM and 3DSM to prepare feeder cells on a large scale in this study. However, how feeder cells maintain the self-renewal capacity and pluripotency of hiPSCs remains unclear and further studies are required to elucidate these issues.

## Conclusion

In summary, we successfully established SAM and 3DSM for the preparation of feeder cells for hiPSCs culture. In SAM, MEF growth is fully inhibited by MMC which is essential for hiPSCs cultures using MEFs as feeder cells. Feeder cells prepared by SAM and 3DSM possess a high direct adherent and recovery rate, and show a high quality in supporting hiPSCs. To increase the processing efficiency and reduce costs, 3DSM was developed to prepare the feeder cells and high processing efficiency of healthy cells was harvested in a short time. Therefore, 3DSM is a more economical way to generate feeder cells for large-scale production of hiPSCs.

## Electronic supplementary material

Supplementary Information
