# Supplementary material for: Efficient feeder cells preparation system for large-scale preparation and application of induced pluripotent stem cells
Source: Sci Rep. 2017 Sep 25;7:12266. doi: 10.1038/s41598-017-10428-5 (PMC5612988; doi:10.1038/s41598-017-10428-5)
Supplement: Supplementary file 1 — Supplementary Information [file 41598_2017_10428_MOESM1_ESM.doc]

**Supplementary Material**

**Efficient feeder cells preparation system for large-scale preparation and application of induced pluripotent stem cells**

Pengdong Li, ShichaoWang, Lixiang Zhan, Xia He, Guangfan Chi, Shuang Lv, Ziran Xu, Yuhan Xia, Shuzhi Teng, Lisha Li^*^ and Yulin Li^*^

**
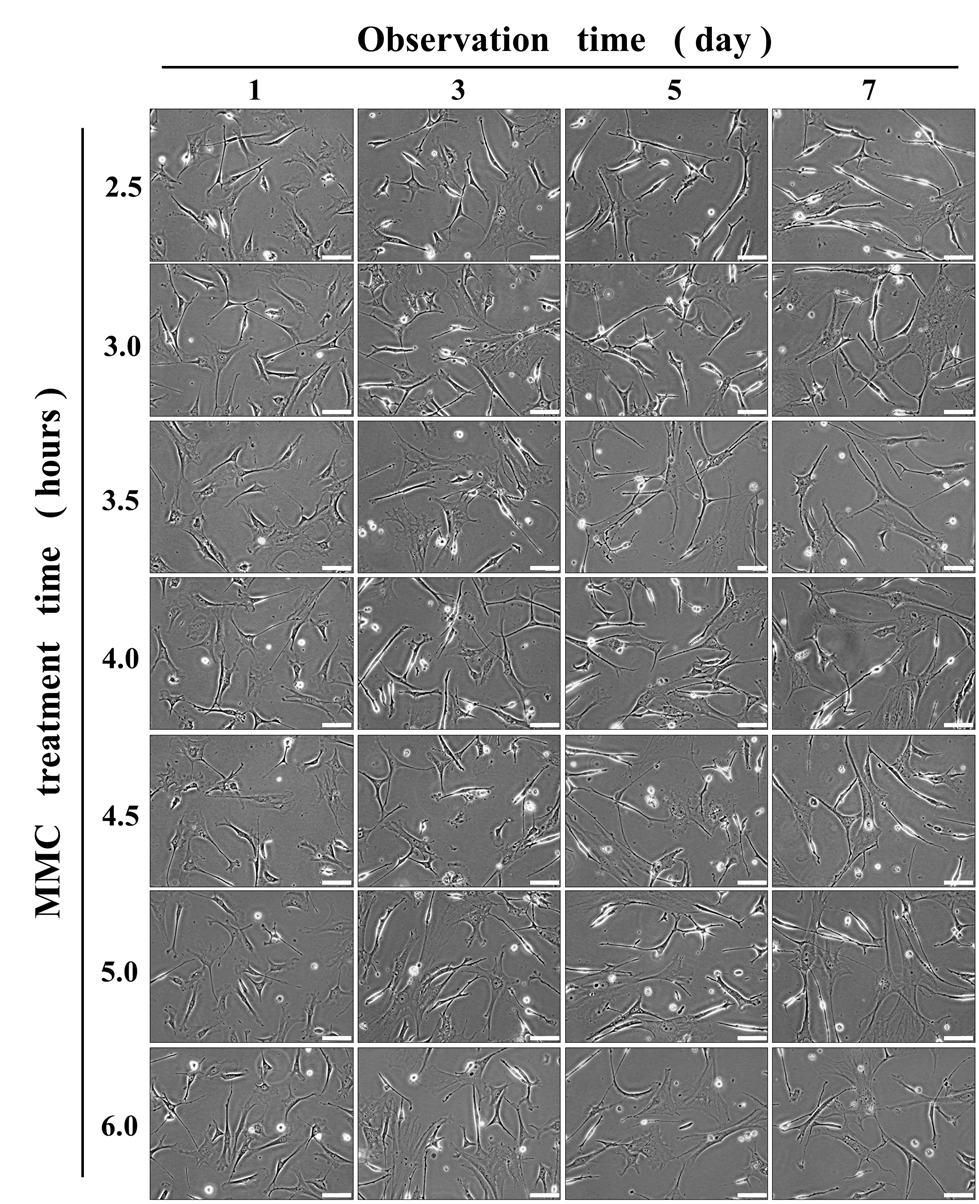
**

Supplementary FIG. S1. Phase contrast microscopy images of MEFs treated with MMC for 2.5, 3.0, 3.5, 4.0, 4.5, 5.0, and 6.0 h in SAM. Bars = 40 μm.

**
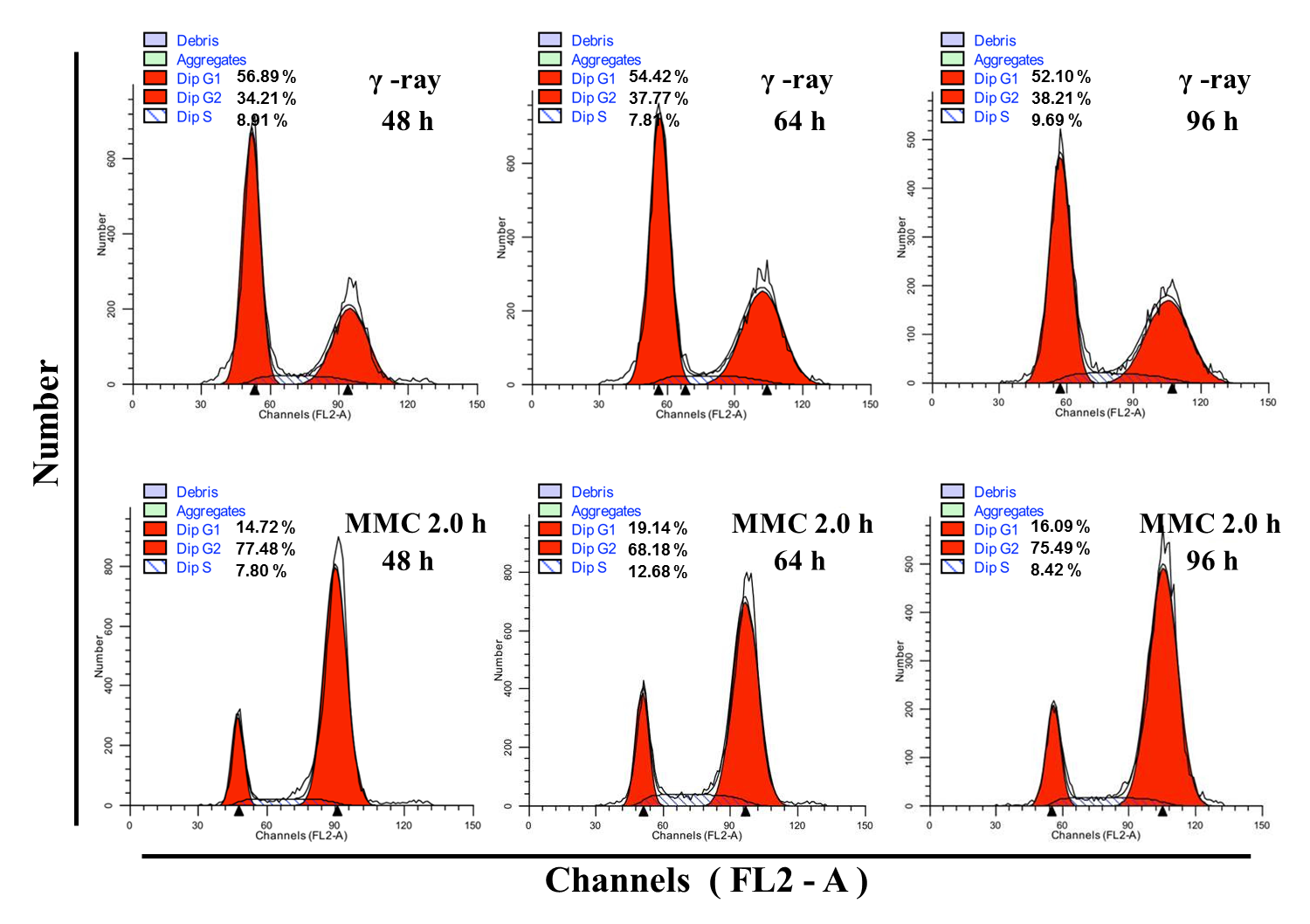
**

Supplementary FIG. S2. Cell cycle analysis of feeder cells treated by CM, SAM, and 3DSM, and cultured for 32, 64, and 96 h. The doubling time of MEFs was about 32 h according to the growth curve (Fig. 3B, 4B and 5B). There is no difference for the percentages of each phase in MMC-treated MEFs among the points. The majority of cells were arrested in the G2 phase after treating with MMC.

Supplementary Table S1. EdU Positive Rates

|  | **Exposure 8 h** | **Exposure 24 h** |
| --- | --- | --- |
| **γ-ray** | 5.11 ± 2.89% | 13.89 ± 5.11% |
| **0 h** | 47.67 ± 5.20% | 65.67 ± 8.28% |
| **0.5 h** | 5.10 ± 2.02% | 14.10 ± 3.14% |
| **1.0 h** | 4.00 ± 1.60% | 15.90 ± 3.45% |
| **1.5 h** | 3.67 ± 2.06% | 13.67 ± 4.09% |
| **2.0 h** | 3.89 ± 2.42% | 14.33 ± 2.06% |
| **2.5 h** | 6.67 ± 4.67% | 16.00 ± 5.79% |
| **3.0 h** | 6.44 ± 2.60% | 14.11 ± 5.04% |
| **3.5 h** | 4.56 ± 2.19% | 13.00 ± 3.32% |
